# The reporting of health systems data use in primary results publications of clinical trials: a systematic review

**DOI:** 10.1186/s13063-025-09227-5

**Published:** 2025-11-26

**Authors:** Jemima Thompson, Marina Bobou, Kate Roberts, Shiva Taheri, Sharon B. Love, Macey L. Murray

**Affiliations:** 1 MRC Clinical Trials Unit at UCL (MRC CTU), Institute of Clinical Trials and Methodology, UCL, London, UK; 2 Health Data Research UK (HDR UK), London, UK

**Keywords:** Health systems data, Routinely collected data, CONSORT-ROUTINE extension

## Abstract

**Background:**

Data collected within clinical trials often overlaps with routinely collected Health Systems Data (HSD). There is potential for HSD to reduce burdens for trials and understanding HSD use can help triallists make decisions about using HSD in future trials. However, it is unknown to what extent HSD use has been reported in trial publications, despite the development of guidelines such as ESMO-GROW and CONSORT-ROUTINE extension for reporting HSD use in trials. This study expands on work previously conducted by Lensen and colleagues (Trials 21(1):398, 2020). It aims to provide insights into how HSD use is reported in main result publications that present main trial results, before and after the release of the CONSORT-ROUTINE extension.

**Methods:**

This was a systematic review of the reporting of HSD use in primary results publications of trials that accessed HSD between 2017 and 2018. Of 90 trials identified by Lensen and colleagues, those that had published primary outcome results were included in the review. Trials were excluded if (1) primary results were not yet due to be reported; (2) not yet published; (3) they were published prior to June 2017; (4) they were published in 2017, but HSD was accessed in 2018 and (5) the primary publication only reported HSD use in secondary, interim or Study Within a Trial (SWAT) analysis. Eligible publications were identified using ISRCTN, ClinicalTrials.gov, EU Clinical Trials Registries, PubMed and Embase. The reporting of HSD use was compared against expectations for reporting outlined in the CONSORT-ROUTINE extension.

**Results:**

Forty-nine primary publications from 46 trials were included in the review**.** Overall, none of the included publications reported all the information suggested in the CONSORT-ROUTINE. However, there has been an improvement in the reporting of HSD use, since the publication of the CONSORT-ROUTINE guidelines.

**Conclusions:**

Reporting of HSD use has improved over time. However, it still does not meet the expectations set out in the CONSORT-ROUTINE extension. Triallists should be encouraged to provide further information in publications about the use of HSD as per the CONSORT-ROUTINE extension guidelines. This would allow greater transparency in reporting, facilitating effective HSD use in future trials.

**Supplementary Information:**

The online version contains supplementary material available at 10.1186/s13063-025-09227-5.

## Background

Health Systems Data (HSD) are formed of routinely collected, longitudinal, real-world data that contain information about patients’ healthcare interactions, such as hospitalisations, mortality and disease occurrence. In the UK, this includes information collected by the National Health Service (NHS), Office for National Statistics (ONS), and disease registries. Traditionally, randomised controlled trials (RCTs) collect data specifically for the purposes of the trial, which is costly in both time and resources [1]. However, some of the information that trials require may already exist within HSD. Therefore, access to HSD may contribute to improving trial efficiency by avoiding duplication of data collection and making the long-term data collection less burdensome for participants and trial staff [1]. Moreover, HSD can be used throughout all stages of a trial, including, feasibility, recruitment, conduct and long-term follow-up [2].

A systematic review, by Lensen and colleagues, identified the characteristics of RCTs that accessed HSD in the UK. They identified clinical trials that had used or planned to use HSD by examining successful data requests to registries. Despite the potential benefits of HSD use, a search of 22 registries revealed that just 3% (*n* = 160) of clinical trials had received HSD between 2013 and 2018 [3]. The authors found that HSD was most often accessed for trials in the disease areas of cancer (29%) and cardiovascular disease (29%). The most common primary outcome examined using HSD was survival and trials that accessed HSD usually had larger sample sizes (median 1590). For 57% of trials, HSD was the sole data source for at least one trial outcome measure, and this was most common in trials using HSD for long-term follow-up. Lensen et al. also reported a lack of transparency relating to the reasons for accessing the data and the datasets that were accessed [3].

Explanations for low uptake of HSD include difficulties accessing databases and registries and issues with data quality [4, 5]. Macnair et al., for example, reported issues with data linkage and encountered several years of delays in gaining permission to access HSD [6]. Furthermore, the utility of HSD may be affected by how frequently databases and registries are updated. Hospital Episode Statistics (HES), for example, can experience delays of several months between hospital attendance and availability of this information in the data asset [2, 7].

Transparency in the reporting of methods used in clinical trials is important for assessing trial quality. It is, therefore, important that information about HSD use, such as rates of data linkage and the adequacy of the data is reported in trial results publications. McCord et al. (2022) suggest that minimum reporting standards should be met in all publications of trials using HSD [8]. However, the reporting of HSD use has previously been shown to be inadequate. In a series of recent reviews, despite the authors describing the advantages of HSD use, important details about HSD use were found to be missing, such as information about validation, data completeness, data linkage and eligibility criteria [9–11]. These inadequacies in reporting lead to a reduced ability for triallists to understand how useful HSD may or may not be as a method of data collection, replicate the methods, assess biases, and understand and interpret trial findings [12]. Improving the reporting of HSD use may provide triallists with information to help support the appropriate uptake of HSD in trials.

To improve the reporting of HSD use, publication checklists have been created; for example, the ESMO–GROW checklist, developed to report the use of real-world data in oncology. The checklist recommends that authors report information such as the type of real-world data used, including information on data linkage processes, data management, governance and metadata [13]. Beyond oncology, an extension to the CONSORT guidelines, CONSORT-ROUTINE, was introduced in 2021 to outline the minimum expectations for reporting HSD use in clinical trials’ main results publications, across disease areas. It includes modifications to existing CONSORT items and the introduction of five new items (ROUTINE 1–5; Table 1) [11]. As outlined by Lensen et al., CONSORT-ROUTINE should be a useful tool for improving transparency in reporting the use of HSD in trial results publications [3]. CONSORT-ROUTINE has been available for almost four years, at the time of writing, but it is not yet known to what extent these reporting guidelines have been implemented and what effect this has had on how HSD is reported in publications of trial results.

**Table 1:** CONSORT-ROUTINE extension items [11]*

Item	Definition
**ROUTINE 1**	Name, if applicable, and description of the cohort or routinely collected database(s) used to conduct the trial, including information on the setting (such as primary care), locations, and dates (such as periods of recruitment, follow-up, and data collection)
**ROUTINE 2**	Eligibility criteria for participants in the cohort or routinely collected database(s)
**ROUTINE 3**	State whether the study included person-level, institutional-level, or other data linkage across two or more databases and, if so, linkage techniques and methods used to evaluate completeness and accuracy of linkage
**ROUTINE 4**	Describe whether and how consent was obtained
**ROUTINE 5**	Information on how to access the list of codes and algorithms used to define or derive the outcomes from the cohort or routinely collected database(s) used to conduct trial, information on accuracy and completeness of outcome variables, and methods used to validate accuracy and completeness (e.g. monitoring, adjudication), if applicable

*Table of CONSORT-ROUTINE extension items table directly obtained from [11]

### Aim

This study aimed to extend the systematic review conducted by Lensen and colleagues [3], and use CONSORT-ROUTINE items 1–5 [11] to examine how HSD use was reported in publications that present main trial results, both before and after publication of the CONSORT-ROUTINE extension.

## Methods

This review was not registered; however, the review protocol is included in the supplementary appendix.

### Inclusion and exclusion criteria

Included the primary results publications of the trials previously identified by Lensen et al. that accessed HSD between 1st January 2017 and 31 st December 2018 (90/160 trials) [3]. Primary results publications were defined as publications that reported results relating to the primary trial outcomes. The 2017–2018 time period was chosen to allow adequate time for the included trials to publish their primary results.

Trials were excluded if the primary results were (1) not yet due to be reported; (2) not yet published; (3) published prior to June 2017; (4) published in 2017 but HSD was accessed in 2018, signifying that HSD was not used in the primary publication; and (5) the primary publication only reported HSD use in secondary, interim or Study Within a Trial (SWAT) analysis (such as Quality of Life, recruitment and other sub-studies). As we considered only primary publications for inclusion in this review to align with the purpose of CONSORT-ROUTINE guidance, this study focused on HSD for within-trial follow-up information (e.g. primary outcomes, secondary outcomes and safety monitoring). Trials that reported using HSD solely to identify potentially eligible patients were, therefore, also excluded.

### Trial selection

Ninety trials were identified that received HSD between 2017 and 2018 [3]. To determine which of these trials were due to report primary outcome results, two members of the review team (MB and JT) independently searched the ISRCTN, ClinicalTrials.gov and EU Clinical Trials Registries. If trials were due to report, but the results articles were not posted, the trial name and acronym were searched in PubMed and Embase, and potential primary publications were screened for inclusion. The two reviewers (JT and MB) compared their publication searches for each of the 90 trials, and discrepancies were discussed. Two further authors were approached (SBL and MLM) if agreement was not reached.

### Data extraction and analysis

Data was extracted and analysed from both the main text of the publications and attached supplementary materials. The main text and supplementary materials of the primary publications were examined to identify whether information relating to HSD access, data quality and HSD cost was reported. Additionally, the main text and supplementary materials of the publications were examined to determine which of the CONSORT-ROUTINE items [1–5], and sub-items, were fulfilled [11]. As we are particularly interested in the methods used to access the accuracy and completeness of HSD, we also considered data utility assessments conducted as a sub-item of routine 5 item [2, 14]. The main text of each publication was read in full to identify information about the use of HSD. Supplementary materials were searched using specific search terms (available in supplementary materials Table 1) [11]. During the searches, it was noted that some data providers have changed names through the years (e.g. NHS Digital changing to NHS England Digital). Therefore, for consistency, their current names will be used throughout.

To ensure consistency in data extraction, two calibration rounds of randomly chosen publications were completed with all six members of the review team. Data extraction discrepancies were discussed until consensus was reached. A digital data extraction template was then created using MS Forms within the Microsoft Office toolkit. The team discussed the development of the data extraction form and relevant updates were made.

All data was double extracted, and consensus discussions were organised between mixed pairs of reviewers. Unresolved queries between pairs were brought to the wider research team. Data were analysed using Stata version 18.5 [15]. Summary statistics are presented as absolute frequencies and percentages.

## Results

Agreement for identification of primary outcome publications meeting the inclusion criteria was 91% (*n* = 82/90 trials) between MB and JT. For the remaining eight trials, agreement was reached through discussion.

### Overview

**Fig. 1:**
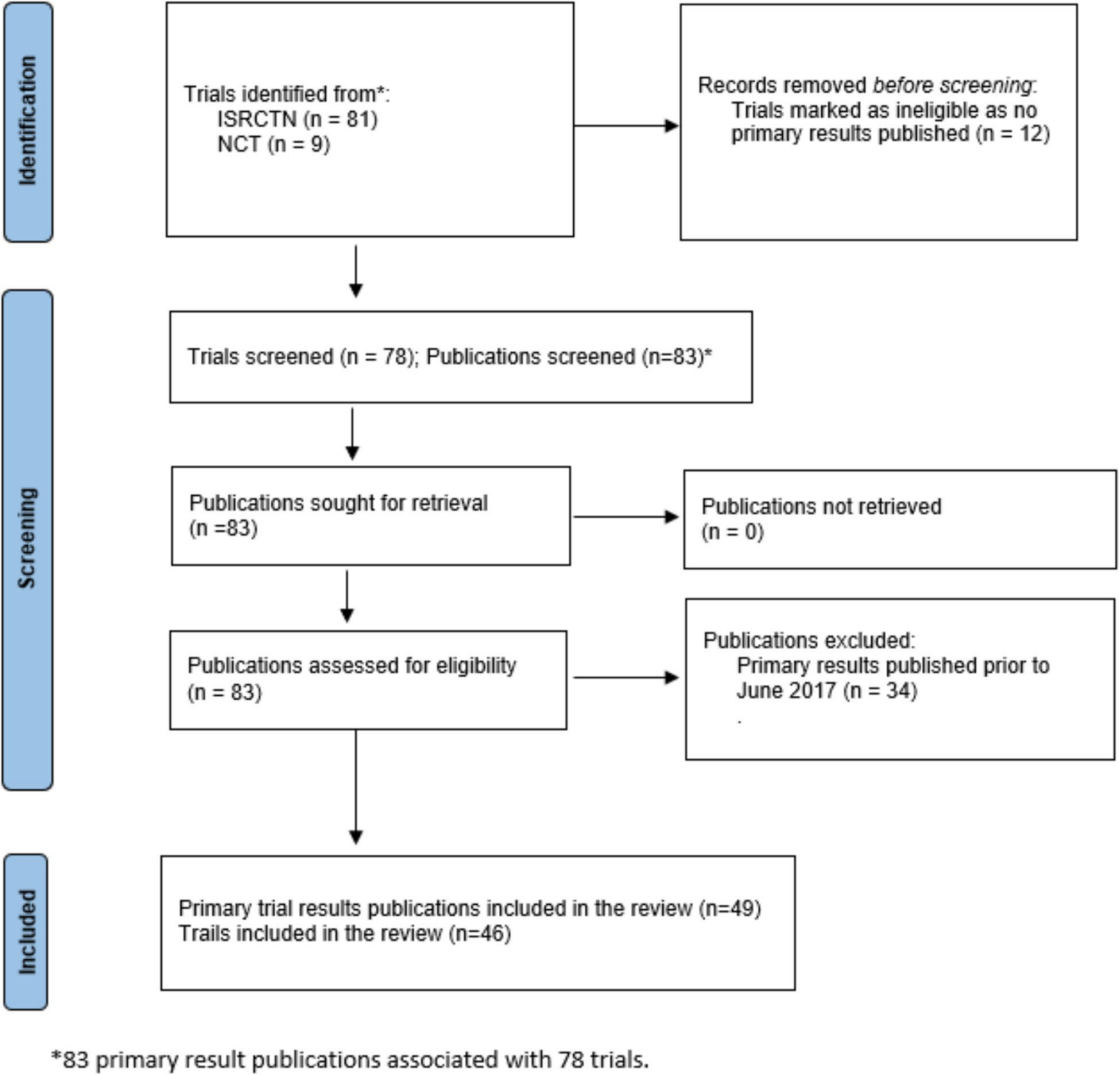
Adapted PRISMA 2020 Flow Diagram for systematic reviews, including searches of databases and registers only

The search for relevant publications was conducted in accordance with PRISMA guidelines (Fig. 1) between June and July 2024. Forty-nine publications were reviewed, presenting results for 46 trials. This was because three trials had two main results publications as they had more than one intervention or primary outcome.

### Trial and publication characteristics

Trial characteristics are presented in Table 2. Most trials (89%; 41/46) took place only in the United Kingdom (UK). The remaining five (11%, 5/46) also included sites from outside the UK. A small percentage were feasibility or pilot trials (15%, 7/46). Most trials used individual randomisation (83%, 38/46) with the remaining trials being cluster randomised (17%, 8/46). The most common disease areas were cardiovascular disease (41%, 19/46), followed by cancer (22%, 10/46). Thirty-nine percent (18/46) of the interventions were of Investigational Medicinal Products.

**Table 2:** Trial characteristics

Trial Characteristic	*n* (%) *n* = *46*
**Location**	
UK only	41 (89%)
International	5 (11%)
**Pilot-Feasibility**	
Yes	7 (15%)
No	39 (85%)
**Randomisation**	
Individual	38 (83%)
Cluster	8 (17%)
**Design**	
Treatment	37 (80%)
Primary prevention	7 (15%)
Screening	2 (5%)
**Blinding**	
Open	35 (76%)
Research staff and patients	8 (17%)
Research staff only	3 (6%)
**Setting**	
Primary care	8 (20%)
Secondary care	37 (80%)
**Intervention**	
Drug	18 (39%)
Surgical	6 (13%)
Other	22 (48%)
**Disease category**	
Cancer	12 (12%)
Cardiovascular/stoke	14 (30%)
Endocrine and diabetes	1 (2%)
Infection	3 (6%)
Inflammatory diseases	2 (4%)
Mental Health	5 (11%)
Pregnancy and childbirth	1 (2%)
Other	8 (17%)
**Primary outcome**	
Survival related	18 (39%)
Other	28 (61%)
**Primary outcome type**	
Clinical	42 (91%)
Administrative	4 (9%)
**Year of publication**	
2017	6 (13%)
2018	5 (11%)
2019	12 (26%)
2020	9 (20%)
2021	3 (6%)
2022	8 (17%)
2023	4 (9%)
2024	1 (2%)
**Publication**	
BMJ	1 (2%)
BMJ Open	2 (5%)
JAMA	4 (9%)
JAMA Network Open	1 (2%)
Lancet	14 (30%)
Lancet – specialities	6 (13%)
NEJM	7 (15%)
Health Services and Delivery Research	1 (2%)
Health Technology Assessment	3 (6%)
Other	9 (20%)

### Location of HSD reporting

**Fig. 2:**
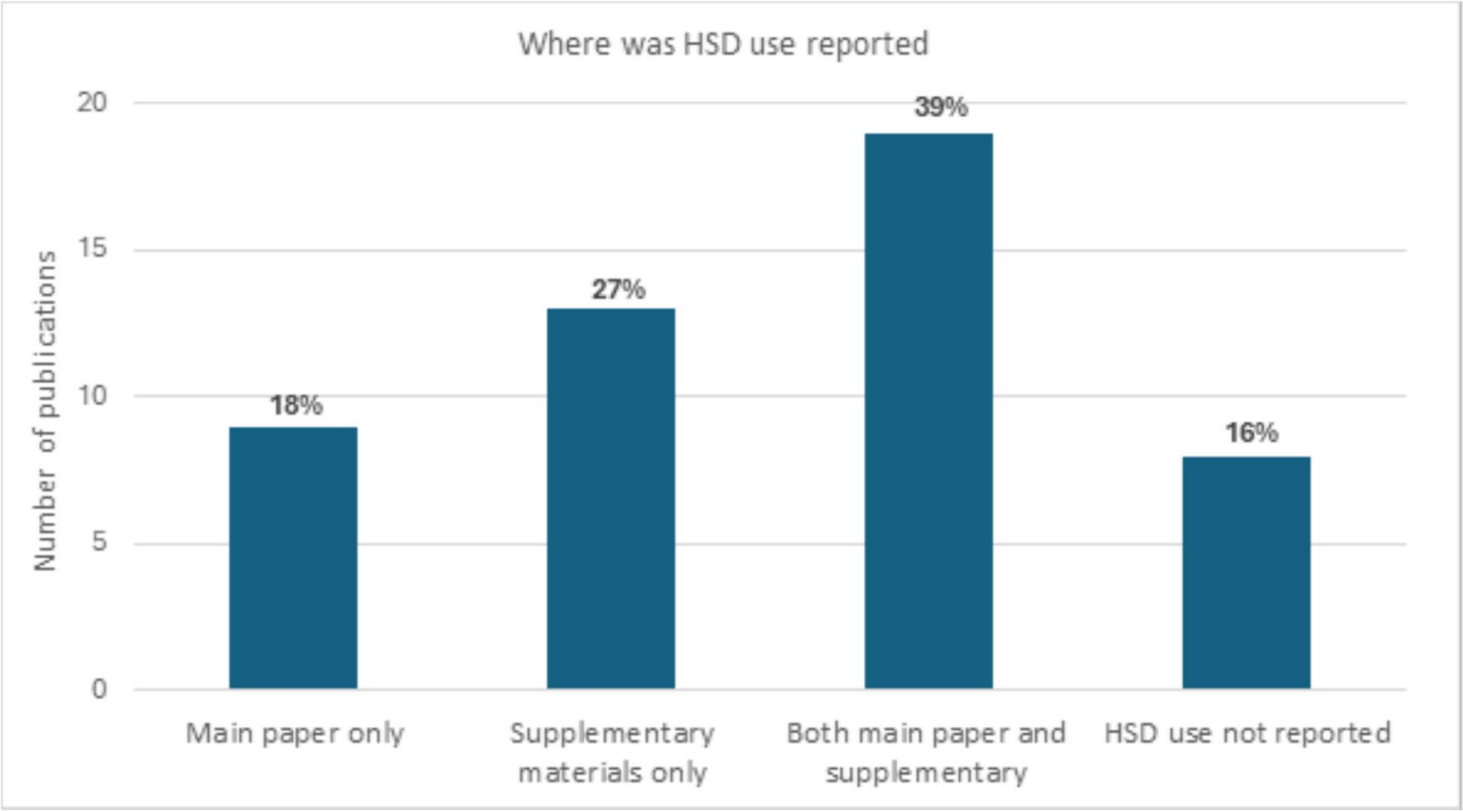
Where HSD use was reported between the main paper and supplementary materials

Figure 2, below, shows how information about HSD use was distributed within the included publications. Information about HSD use was most often reported in a combination of the main text and supplementary materials (39%, 19/49) [16–34]. Six publications (12%, 6/49) did not include any information about HSD use [35–42].

### Challenges with HSD access, data quality and cost

Issues obtaining access to HSD were reported in six (12%, 6/49) publications [20, 21, 28, 31, 43, 44]. Issues primarily related to delays in gaining access to the relevant datasets, caused by changes in Information Governance over the course of the trial [20], extended discussion and correspondence [28, 44], and problems created by the COVID-19 pandemic [31]. The REDUCE trial publication reported that some GP surgeries migrated to new systems, so some data could not be collected [43]. The FAST trial reported being unable to obtain data linkage in Sweden, though the reasons for this were not explicitly explained [21].

Data quality issues were reported in two (4%, 2/49) publications. For the EPOCH trial, there was no specific International Classification of Diseases (ICD) code to capture the primary outcome event of interest (emergency abdominal surgery); thus, ICD codes pertaining to “relevant procedures” were used [20]. In the SEND trial, authors report that just 3% of Emergency Department visits found in HSD matched the number that was self-reported by trial participants [38]. There were no issues reported regarding the cost of HSD access in any of the 49 trial publications.

### Overview of CONSORT-ROUTINE items

**Fig. 3:**
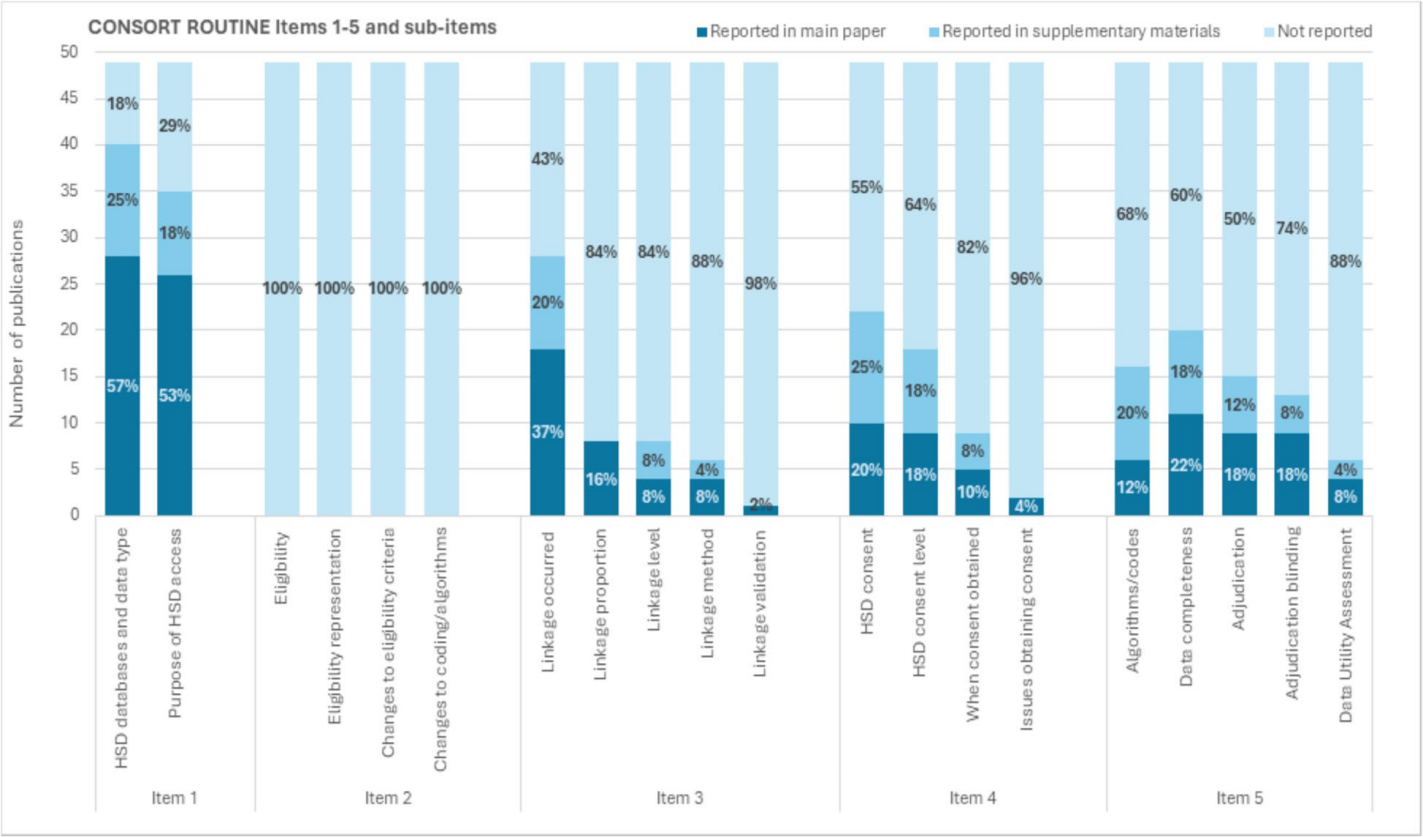
Reporting of CONSORT-ROUTINE items 1–5 and sub-items in main papers and supplementary materials

Figure 3 summarises the reporting of CONSORT-ROUTINE items 1–5 in the main paper and supplementary materials of each publication. There are variations evident in what aspects of HSD use have been reported in the included publications. Sub-items of item 1 are reported in most publications, whilst sub-items within items 3, 4 and 5 are reported less frequently. None of the sub-items of item 2 were reported in any of the publications.

#### Reporting of item 1—cohort or routinely collected database

Item 1 suggests that authors explicitly state the type of HSD accessed (e.g. NHS England Digital or disease registries) and the purpose of the access, such as for primary outcome measurement or identification of safety events [11]. The HSD accessed were reported in 40 (82%, 40/49) of the trial publications (for details see supplementary materials Table 1). The purpose of HSD access was reported in approximately three-quarters of the publications (71%, 35/49) [16–22, 25–28, 30–34, 39, 43–59].

#### Reporting of item 2—eligibility criteria

Item 2 suggests that authors should give a description of the entry criteria into the cohort or routinely collected database and explain whether this may affect the sample population [11]. Though trial eligibility criteria were presented in most publications, explicit reporting of the effects of eligibility on information collected using HSD was not present. Therefore, this item was not fulfilled in any of the included publications.

#### Reporting of item 3—HSD linkage

For item 3, authors were expected to report information about data linkage, including the proportion of successfully linked participants, linkage level (e.g. personal or institutional), linkage methods (e.g. direct using unique identifiers, such as NHS number, or indirect, using identifiers such as region or postcode or probabilistic methods) and whether any validation was performed [11]. More than half of the included publications reported that data linkage occurred (59%, 29/49) [16, 17, 19, 21, 24–28, 30–34, 39, 43, 44, 47–49, 51, 54–58, 60, 61]. The proportion of successfully linked participants was reported in eight (16%, 8/49) publications, with all these trials achieving a successful linkage rate of 96% or above [19, 28, 29, 33, 44, 47, 51, 62]. The level of linkage was reported in eight (16%, 8/49) publications [27, 28, 30, 32, 34, 44, 48, 54], and the method of linkage was reported in seven (14%, 7/49) publications [27, 28, 30, 32, 44, 48, 54].

#### Reporting of item 4—consent

Item 4 suggests that authors explain whether consent to access HSD was obtained institutionally or from participants, which will depend on whether the trial is individually, or cluster randomised. Authors should also report when consent was obtained and if any issues were encountered [11]. Consent for HSD use was mentioned in 22 (45%, 22/49) publications [16, 17, 19–21, 25–28, 31, 33, 34, 39, 43, 44, 48, 51, 54–58]. The level of consent was reported in 16 (33%, 16/49) publications [18, 24, 25, 30–33, 39, 41, 43, 44, 51, 52, 55, 57, 58]. The timing of the consent to access HSD from participants was reported in nine (18%, 9/49) publications [19, 27, 28, 33, 41, 43, 44, 51, 56]. Two publications explicitly reported issues with obtaining consent to access HSD (4%, 2/49).

#### Reporting of item 5—algorithms, completeness, adjudication and data utility

For item 5, authors are expected to provide the coding algorithms used to define outcomes, describe the completeness of the HSD, specify whether adjudication took place and whether this was blinded to trial allocation, and report whether any data utility assessments were performed [11]. A third of publications reported using algorithms or codes to identify trial outcomes (32%, 16/49) [16, 17, 19–21, 25, 26, 28, 31, 32, 34, 43, 46, 52, 53, 61]. Data completeness was reported in 20 (41%, 20/49) publications [16–20, 22–25, 28, 30, 33, 44–46, 50]. Adjudication procedures were reported in almost a third of publications (31%, 15/49) [17, 19, 21, 25–28, 31, 32, 39, 45, 46, 52, 53, 57] and around a quarter of publications reported whether adjudicators were blinded (27%, 13/49) [17, 19, 21, 25, 26, 28, 31, 39, 45, 46, 52, 53, 57]. Just six publications (12%, 6/49) reported assessing data utility [21, 23–25, 49, 57].

#### Total Item completion (CONSORT-ROUTINE items 1–5)

**Fig. 4:**
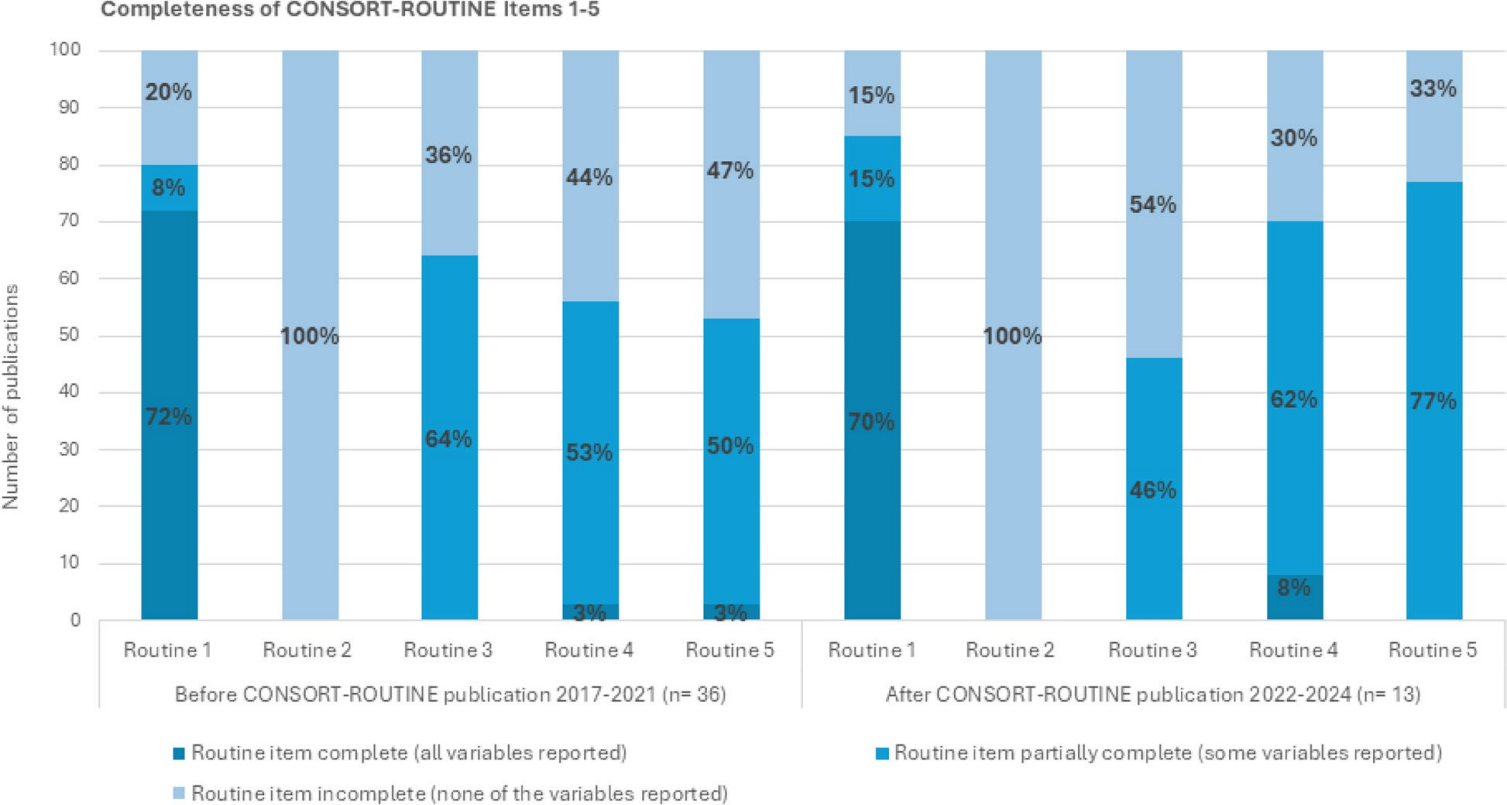
Proportion of complete CONSORT-ROUTINE items

For each item to be considered reported completely, all sub-items included within the item needed to be reported in the publications. The completeness of each CONSORT-ROUTINE item is outlined in Fig. 4. This figure divides the included publications based on whether they were published before the release of the CONSORT-ROUTINE extension guidelines (2017–2021) or after (2022–2024). Item 1 was complete in around three-quarters of publications (71%, 35/49) [16–22, 25–28, 30–34, 39, 43–59]. Items 4 and 5 were rarely completed, with just two (4%, 2/49) of publications complete for item 4 [27, 33] and one (2%, 1/49) for item 5 [25]. For items 1, 3, 4 and 5, several publications reported on some sub-components of each item. As previously outlined, item 2 was not complete for any of the 49 publications. The proportion of complete and partially complete items improved slightly from 2022 onwards for items 1, 4 and 5, but decreased slightly for item 3.

## Discussion

This review examined how HSD use was reported in 49 primary outcome results publications of 46 UK-based trials that received HSD between 2017 and 2018. Findings from this review demonstrate some signs of improvement in the reporting of HSD use. There appears to have been a small increase in reporting on cohorts and datasets (item 1), consent (item 4), and data utility (item 5) over time, but the reporting of eligibility (item 2) remains unchanged, and the detailing of data linkage (item 3) shows a slight decline.

Most of the publications included in this review reported HSD use, in some capacity (88%, 43/49). Explanations for why HSD use was not reported, despite HSD being received, was only provided in one of the remaining (12%, 6/49) publications. In the BRIDGE-IT publication [60], the authors explained that HSD was to be used during long-term follow-up, not for the primary outcome which was being reported in this publication. Explanations for the absence of reporting, such as that provided in the BRIDGE-IT trial publication [60], would be useful for highlighting where access to HSD has been planned but not used.

Much of the information relating to HSD use was reported in supplementary materials. This could be attributed to word count restrictions from high-impact journals, which can make decision-making about which information to include in the main text difficult for authors. However, though not explicitly stated in the CONSORT-ROUTINE extension, the CONSORT checklist indicates that most information required for the publication of trial results should be reported in the main text.

Though the main body of the text is a reasonable place to report most aspects of HSD use, it is acknowledged that some details about its use, such as long lists of diagnostic codes and algorithms (such as those expected to be reported in item 5), may be best situated within the supplementary materials. In some publications, information about HSD use was reported in an attached trial protocol. Whilst it is a requirement for some journals (such as the Lancet) to include a trial protocol in the supplementary materials, we suggest that caution be exercised when discussing HSD use reported in trial protocols, as it cannot be assumed whether the proposed plans materialised. This is also a key reason not to examine HSD use based on published protocol papers or interim publications.

Despite known issues with gaining access to HSD [4–6, 63] and concerns about the quality of HSD and its utility, challenges in access and quality of HSD were rarely reported. Like findings from Imran et al. [63], issues with the cost of HSD access were not reported in any of the 49 publications. However, the omission of such information does not mean that trials did not experience challenges or concerns relating to HSD access, data quality or cost. Explicitly stating whether problems were encountered, increases transparency in reporting, meaning that future trials can implement design mitigations to address known problems, leading to more efficient and reliable data collection. It is of note that most of the trials included in this review took place exclusively in the UK. However, differences in the healthcare systems across the four UK nations, and in other countries around the world should be considered.

There was considerable variation in how much and what information about HSD use was reported across the five CONSORT-ROUTINE items. Most publications included information that aligned with expectations for reporting in item 1 (cohort and databases). However, some publications did not report which databases they used to access, an observation that raises questions as all of the trials included in this review accessed HSD [3]. Strikingly, information required to meet expectations for item 2 (eligibility) was not reported in any of the 49 included publications. Trial publications usually reported the eligibility criteria for the trial, either in the main text or in the trial protocol. However, how the HSD databases and registries could impact the sample was not acknowledged. Over time, the information stored on HSD databases and registries can change, as can trial eligibility criteria. By explicitly stating exactly what data is being collected and whether changes to the databases/eligibility criteria have occurred, clearer assessments can be made about the data collected and its reliability and completeness. Rates of reporting were generally low across items 3, 4 and 5. A possible explanation for the inconsistencies found within item 3 (linkage) and item 4 (consent), is that linkage [55] and consent procedures can vary greatly when the trial is cluster randomised rather than individually randomised.

These findings differ from results presented in Imran et al.’s [63] review of 33 trial publications, published between 2011 and 2018. Though Imran and colleagues found that reporting of HSD was inadequate in over 50% of the included publications, similar to the current review, the way that information was reported across the five CONSORT-ROUTINE items differed. Imran et al. reported item 4 (informed consent) as being the most adequately completed item and item 2 (eligibility) was inadequately reported 75% of the time. Differences between Imran et al. review and the current review could be explained by differences in publication dates and the locations of the trials being reported. Trials included in Imran et al. review primarily took place in the USA and Canada, who may have different registries and access agreements in place when compared to trials operating from the UK. Imran et al. included both primary outcome publications and secondary publications in their review. Furthermore, in the current review supplementary materials were included in the data extraction, which did not appear to be the case in Imran et al.’s review.

Findings from this review suggest that whilst some improvements have been made in the reporting of HSD use in trial results publications, there is still room for improvement. This could indicate either that the CONSORT-ROUTINE guidance has yet to be consistently implemented, that the guidance is not as clear as it needs to be, or a combination of both.

Further clarity around the reporting of eligibility criteria for the use of HSD, in particular, needs to be addressed. Additional transparency in reporting around issues such as cost and time to access, also mean that this information can be provided to funders and be accounted for more explicitly when considering trial design, allocation of trial budgets and the timings of applications to access HSD. Whilst CONSORT-ROUTINE is potentially a useful tool for improving reporting transparency, some aspects of the guidelines are not always explicit or clear about exactly what should be reported.

### Future recommendations

1. We suggest that funders make trial teams aware of the expectations set out within CONSORT-ROUTINE guidelines, so that they can be used to inform the design, allocations of trial funding and the timing of HSD access applications in trials that plan to use HSD for data collection purposes.
2. We suggest that more journal editors could consider incorporating CONSORT-ROUTINE guidelines into the author instructions, which will support clearer reporting of HSD use.
3. Authors using HSD in trials might benefit from more detailed guidance with further examples or use cases. Thus, an update to the CONSORT-ROUTINE is suggested, rendering data collection and reporting more efficient and reliable.

### Strengths and limitations

The search for trial publications and subsequent data extraction involved multiple reviewers, making the process robust and reducing potential for bias. However, this review does have some limitations. Most publications reviewed were published prior to the publication of the CONSORT-ROUTINE extension, when the reporting expectations for HSD use may have been less clear for authors. Additionally, when selecting the trials for this review, we were only aware that the included trials had received HSD, not how they intended to use it. This review focused exclusively on primary results publications. Therefore, in trials using HSD to inform other outcomes (i.e. secondary, long-term follow up, feasibility etc.) it may have been more appropriate to report HSD use elsewhere.

## Conclusion

This review has examined the reporting of HSD use in trial primary outcome publications. Although some aspects of HSD use are reported, and this has slightly improved since the introduction of CONSORT-ROUTINE, a considerable amount of essential information about HSD use is still not being reported. More clarity about the importance of reporting HSD is required to ensure that authors are aware of expectations. Updates to the current CONSORT-ROUTINE guidance, and support from research funders and journal editors to ensure that CONSORT-ROUTINE guidance is followed are therefore recommended to ensure greater transparency in reporting and facilitate the effective use of HSD in future trials.

## Supplementary Information

Additional file 1. Supplementary Material.

## Data Availability

The datasets used and/or analysed during the current study are available from the corresponding author on reasonable request.
