# Supplementary material for: The reporting of health systems data use in primary results publications of clinical trials: a systematic review
Source: Trials. 2025 Nov 26;26:549. doi: 10.1186/s13063-025-09227-5 (PMC12659570; doi:10.1186/s13063-025-09227-5)
Supplement: Supplementary file 1 — Additional file 1. Supplementary Material. [file 13063_2025_9227_MOESM1_ESM.docx]

**Supplementary Table 1:** Item 1 sub-item purpose of HSD access (n= 49)*

| **Purpose** | **N (%)** | **References** |
| --- | --- | --- |
| Primary Outcome | 21 (45%) | 16, 17, 19-21, 25-28, 31-33, 35, 43, 44, 46, 47, 51, 52, 55, 58 |
| Secondary Outcome | 15 (31%) | 17, 19-22, 27, 31-33, 43, 46, 49, 52, 58, 59 |
| Long-term Follow Up | 10 (20%) | 18, 30, 39, 44, 50, 52-54, 56, 59 |
| Safety outcomes | 9 (18%) | 17, 18, 22, 29, 33, 43, 45, 48, 57 |
| Recruitment | 1 (2%) | 48 |
| Other | 4 (8%) | 32, 47, 59, 61 |

*Purpose categories are not mutually exclusive, as use HSD for more than one purpose

**Supplementary Table 2:** Item 3 sub-items linkage level and linkage method

| **Linkage sub-item** | | **N (%)** | **References** |
| --- | --- | --- | --- |
| Linkage Level | Personal | 7 (14%) | 27, 28, 30, 32, 44, 48, 54 |
|  | Institutional | 1 (2%) | 35 |
| Linkage Methods | Direct | 6 (12%) | 28, 30, 32, 44, 48, 54 |
|  | Indirect | 1 (2%) | 27 |

**Supplementary Table 3:** Item 4 sub-item consent level

| **Consent level** | **N (%)** | **References** |
| --- | --- | --- |
| Individual | 13 (27%) | 17, 19, 27, 28, 31, 33, 39, 44, 48, 54-56, 58 |
| Institutional | 3 (6%) | 20, 35, 43 |

**Supplementary Table 4:** Item 4 sub-item consent issues

| **Publication reference** | **Issue reported** |
| --- | --- |
| VIDAL ^(44)^ | The wording of the informed consent forms needed to be revised, despite having been reviewed and approved by their patient representatives and senior staff members. |
| REDUCE ^(43)^ | Consent was not obtained to perform analysis using the entire Clinical Practice Research Datalink (CPRD) dataset. |

**Supplementary Table 5:** Item 5 sub-item data utility reporting.

| **Publication reference** | **Data Utility report** |
| --- | --- |
| FOXTROT ^(45)^ | Reported discrepancies between HSD and trial data |
| VIDAL ^(44)^ | Reported discrepancies between HSD and trial data |
| AFFINITIE ^(35)^ | Completed a parallel process evaluation |
| IMPROVE ^(51)^ | Reported checking the completeness of trial data against HES data. |
| PreFIT ^(25)^ | The authors explain in trial protocol they were planning to explore which of their proposed data sources would be most accurate (Hospital Episode Statistics and GP records) during the feasibility phase of the trial. |
| UKLS ^(33)^ | Authors mention in trial protocol that there may be missing data for the reporting of primary and secondary outcomes but that no formal checking would be done for this. |
